# Nanowire systems: technology and design

**DOI:** 10.1098/rsta.2013.0102

**Published:** 2014-03-28

**Authors:** Pierre-Emmanuel Gaillardon, Luca Gaetano Amarù, Shashikanth Bobba, Michele De Marchi, Davide Sacchetto, Giovanni De Micheli

**Affiliations:** EPFL, Lausanne, Switzerland

**Keywords:** nanosystems, nanoelectronics, nanowire transistors, controllable polarity, regular arrays, logic synthesis

## Abstract

Nanosystems are large-scale integrated systems exploiting nanoelectronic devices. In this study, we consider *double independent gate, vertically stacked nanowire field effect transistors* (FETs) with gate-all-around structures and typical diameter of 20 nm. These devices, which we have successfully fabricated and evaluated, control the ambipolar behaviour of the nanostructure by selectively enabling one type of carriers. These transistors work as switches with electrically programmable polarity and thus realize an *exclusive or* operation. The intrinsic higher expressive power of these FETs, when compared with standard *complementary metal oxide semiconductor* technology, enables us to realize more efficient logic gates, which we organize as tiles to realize nanowire systems by regular arrays. This article surveys both the technology for double independent gate FETs as well as physical and logic design tools to realize digital systems with this fabrication technology.

## 1. Introduction

*Nanosystems* are integrated systems exploiting *nano-* *electronic* devices. Extreme miniaturization has multiple positive effects, including better electronic properties (e.g. performance) and lower cost. In particular, this work considers *silicon nanowire* (SiNW) technology as a possible replacement/enhancement of current device technologies and design issues for integrated *nanowire systems*. The interest in exploring new technological approaches to very-large-scale *system-on-chip* (SoC) design stems from the physical limitations and the costs of current manufacturing technologies and from the desire to use more efficient devices, still within the realm of silicon manufacturing. The downscaling of the physical features of *field-effect transistors* (FETs) has successfully produced better and cheaper devices. Nevertheless, current semiconductor technologies have succeeded mainly along two avenues: *fully depleted silicon on insulator* [1] and *FinFET* [2,3] technologies. The latter (also called TriGate technology) is a major departure from planar semiconductor manufacturing: better transistor charge control is achieved at the price of a more complex three-dimensional fabrication process. Within the quest of future technologies, we describe here *vertically stacked silicon nanowire field effect transistors* (SiNWFETs) [4] as a promising extension to the FinFETs.

An *SiNW* is a thin wire of silicon material, with a diameter ranging from some nanometres to some tenths of nanometres. Transistors are formed by surrounding a segment of the wire by an insulator (such as SiO_2_ or HfO_2_) and then by a coaxial conducting material (gate), thus forming a so-called *gate-all-around* (GAA) transistor. This structure yields an excellent electrostatic control of the transistor channel, consisting of the nanowire itself under the gate. As a measurable result, the transistor gives a higher *I*_on_/*I*_off_ ratio (i.e. current ratio of the conducting over non-conducting device) [5].

At advanced technology nodes, an increasingly larger number of devices are affected by Schottky contacts at the source and drain interfaces. Hence, devices face an ambipolar behaviour, i.e. each device exhibits *n*- and *p*-type characteristics simultaneously because of the possible flow of electrons and holes in the channel. This phenomenon is often suppressed in most technologies because of the desire to create unipolar transistors, i.e. devices with a specific type of carriers: electrons for *n*-type and holes for *p*-type transistors. Nevertheless, an important recent breakthrough has shown [6,7] that it is of high interest to control the ambipolar phenomenon through *programmable polarity* devices. Indeed, by engineering the source and drain contacts and by constructing independent *double-gate* (DG) structures, the device polarity can be electrostatically programmed to be either *n*- or *p*-type at run time. The functionality of a transistor with controllable polarity is an *exclusive or* (EXOR) of the logic signals on both gates. Thus, the fundamental switching primitive, the DG-SiNWFET, is intrinsically more expressive in terms of logic when compared with standard *complementary metal oxide semiconductor* (CMOS) transistors. In other words, while regular transistors act as switches, the DG-SiNWFETs act as comparators.

The potential advantage of this powerful logic primitive may be offset by the interconnect complexity. This trend is not a surprise for nanosystems in general, including scaled CMOS. Regularity is one of the key features to increase the yield of integrated circuits at advanced technology nodes [8], while keeping the routing complexity under control. Therefore, nanowire systems can be realized as regular arrays of elementary logic blocks, called *sea of tiles* (SoT) [9]. Thanks to a novel symbolic layout methodology, a desired logic function can be mapped onto an array of logic tiles, thereby enabling the automatic placement of digital circuits onto a SoT organization.

In a similar vein, a logic design has to be mapped efficiently onto the SiNW primitives. These primitives can support the realization of both unate and binate functions. Note that CMOS logic primitives are inherently inverting, thus privileging the realization of negative unate functions. Hence, logic synthesis and algorithms supporting the mapping of architectural-level specification into DG-SiNWFET netlists are mandatory.

This paper aims at surveying the main results associated with DG-SiNWFETs from technology to physical design and to logic synthesis. The remainder of the paper is organized as follows. In §2, we present our DG-SiNWFET technology and its circuit-level features. In §3, we introduce means of describing regular transistor arrangements to mitigate the impact of the additional gate, and summarize the associated physical design methodology. In §4, we describe the basis for a new logic synthesis flow, whereas, in §5, we derive the potential of the approach for arithmetic and fault-tolerant architectures. Section 6 concludes this work.

## 2. Technology overview

Here, we introduce the technology of DG-SiNWFETs and the associated circuit structures.

### (a) Transistors with controllable polarity

The ambipolar conduction phenomenon is observable in several nanoscale FET devices (45 nm node and below), including silicon [10], carbon nanotubes [11] and graphene [12]. The control of the ambipolarity allows us to adjust the device polarity online. Such transistors, i.e. with a controllable polarity, have been experimentally fabricated in several novel technologies, such as carbon nanotubes [13], graphene [14] and SiNWs [15,16]. To the best of our knowledge, Sacchetto *et al.* [17] and De Marchi *et al.* [18] were the first to fabricate and test successfully SiNW transistors with independent individual control. They introduced DG-SiNWFETs where one gate controls the polarity (i.e. type of carrier, *n* or *p*), whereas the other gate controls the carrier flow in the channel. The operation of these FETs is enabled by the regulation of Schottky barriers on source/drain junctions through the additional gate.

**Figure 1.**
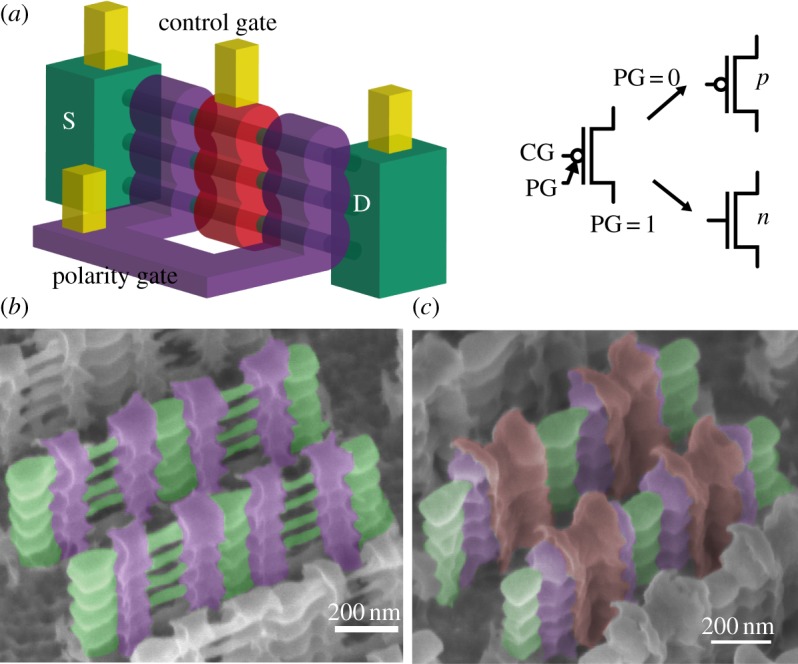
Three-dimensional sketch of the SiNWFET featuring two independent gates and its associated symbol (*a*). Tilted SEM views of an array of fabricated devices before creation of the control gate (*b*) and after addition of the polarity gates (*c*). S/D pillars and nanowires (green), PG (violet) and CG (red) are shown [18].

In particular, De Marchi *et al.* [18] fabricated vertically stacked SiNWFETs, featuring two *gate-all-around* electrodes (figure 1). Vertically stacked GAA SiNWs represent a natural evolution of FinFET structures, providing better electrostatic control over the channel and consequently superior scalability properties [18].

**Figure 2.**
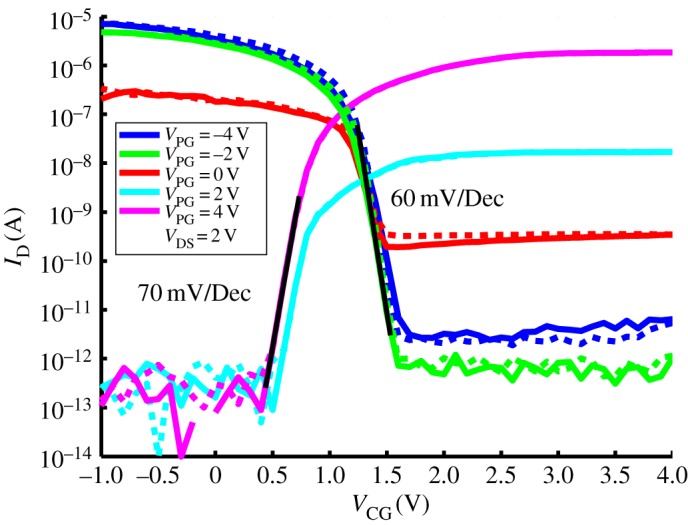
*I*_DS_–*V*_CG_ logarithmic plot of a measured device for several *V*_PG_ voltages. Curves extracted at *V*_DS_=2 V [18].

In the device, one gate electrode, the *control gate* (CG), acts conventionally by turning *on* and *off* the device. The other electrode, the *polarity gate* (PG), acts on the side regions of the device, in proximity of the *source/drain* (S/D) Schottky junctions, switching the device polarity dynamically between *n*- and *p*-type (figure 2). The input and output voltage levels are compatible, resulting in directly cascadable logic gates. It should be noted that owing to the device geometries, the two gates are not identical from a size standpoint. Indeed, the PG is roughly two times bigger than the CG, leading to differences in their timing responses. Such a behaviour can be easily compensated at the design level by assigning the signal with the lowest frequency/switching activity to the slowest gate terminal.

Thanks to their one-dimensional structure, DG-SiNWFETs demonstrate remarkable electrostatic performances. Figure 2 depicts the subthreshold slopes of 64 mV/Dec and 70 mV/Dec for the *p*-type and *n*-type parts of the characteristic, respectively, hence competing with the most advanced FinFET technologies [3]. In addition, the one-dimensional electrostatic control over the channel coupled to the use of a Schottky barrier-based injection mechanism enables very low *off-current* densities of a few pA per μm when compared with few tens of pA per μm for low-power FinFETs [3]. These combined facts qualify the presented device technology as *high-performance low-standby-power* technology.

### (b) Logic operations with double-gate field effect transistors

**Figure 3.**
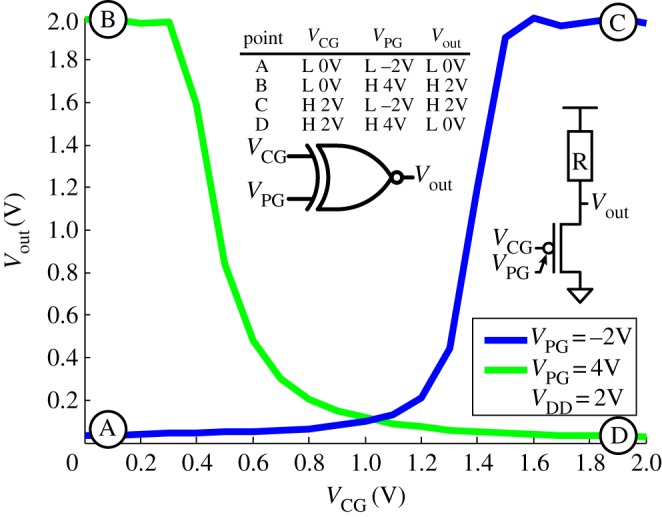
Pseudo-logic XOR characteristic obtained using a single SiNWFET with controllable polarity [19].

*Digital circuits using these transistors can exploit both gates as inputs, thereby enabling the design of compact cells that implement XOR more efficiently than in CMOS*. Indeed, in the context of digital operations, DG-SiNWFETs realize intrinsically an XOR characteristic, because the transistor is ON when *PG*=CG, i.e. 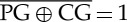, and consequently is OFF when PG⊕*CG*=1. Figure 3 presents a pseudo-logic XOR gate. The device in the *pull-down* network is polarized by means of the PG. In the case of the *n*-type polarization, the characteristics of a pseudo-logic inverter are obtained (green). In the *p*-type polarization, a buffer is obtained (blue). As shown in the inset truth table, an XOR function can be implemented by a single transistor and a pull-up.

**Figure 4.**
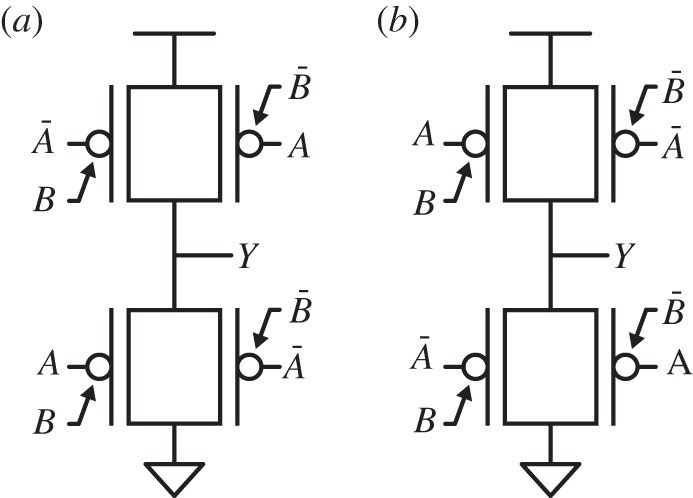
Two-input XOR (*a*) and XNOR (*b*) gates built with DG-SiNWFETs [7].

The unique feature of this device of being polarized electrostatically was first used to build a reconfigurable logic cell [6], and later used to define a static XOR-intensive logic family [7]. In particular, a full-swing two-input XOR gate can be achieved by using a complementary pull-up and parallel transistors to avoid threshold drops. The XOR and XNOR implementations, reported in figure 4, require four transistors, whereas the traditional full-swing static CMOS implementation uses eight transistors [20].

**Figure 5.**
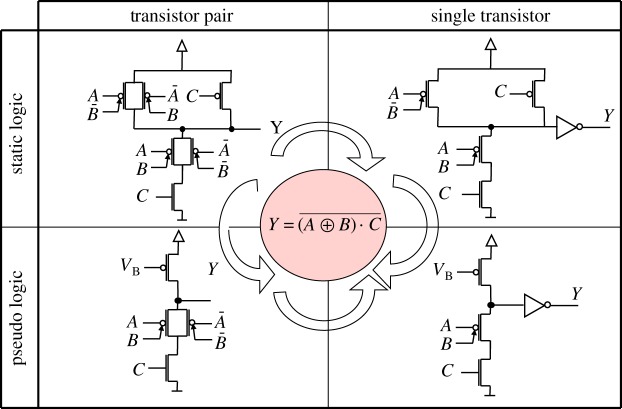
Various implementations of the function 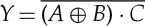. (Online version in colour.)

Various families of logic gates can be designed for DG-SiNWFETs. In particular, one can extend the principle shown in figure 4 to design arbitrary combinational logic functions. Alternatively, fewer transistors can be used by either using a dynamic (or resistive) load, or by correcting the reduced swing owing to threshold drops by using an output buffer. Examples of realizations of arbitrary functions are shown in figure 5.

## 3. Sea of tiles: how to deal with the routing congestion

**Figure 6.**
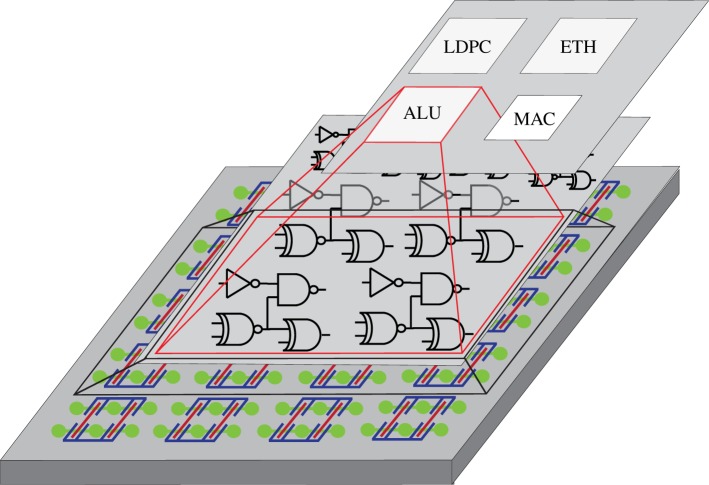
Conceptual representation of a regular *sea of tiles*. Tiles are configured to realize logic functions that are part of a complex system such as a processor [19]. (Online version in colour.)

Regular layout fabrics have the advantage of higher yield as they maximize layout manufacturability. In this section, we describe a novel architecture, called SoT, which is an array of logic tiles that are uniformly spread across the chip. The concept is illustrated in figure 6. Each tile is a template that can be wired to implement an elementary logic gate, such as a NOR, NAND, XOR, DFF or more generally a single-output combinational logic function. Note first that functions realized in ambipolar technology are not restricted to be unate. It is important to stress that the choice of logic tile (or tiles) to use in an array is important, as larger tiles can implement more complex functions, but waste devices for smaller functions, as in the case of gate arrays.

### (a) Towards a regular gate arrangement

Layout regularity is one of the key features required to increase the yield of integrated circuits at advanced technology nodes [8]. Various regular fabrics have been proposed throughout the evolution of the semiconductor industry, with some recent approaches explained in [8,21,22]. In gate-array fabric style, a sea of prefabricated transistors is customized to obtain a desired logic gate. The customization of generic gate arrays comes at a large area cost as well as routing overhead, thereby increasing the performance gap between application-specific integrated circuits (ASICs) and gate arrays. However, strict design rules, at 22 nm technology node and beyond, have led to ASIC cell layouts with arrays of gates with a constant gate pitch, which resemble a sea-of-gates layout style. In Bobba *et al.* [9], a logic *tile* was defined as a fixed pattern of prefabricated transistor pairs grouped together. Uncommitted tiles can then be mapped to logic cells by connecting the gates and the S/D free terminals.

### (b) Layout techniques

To enable the compact implementation of functions with the proposed transistors, we use a novel symbolic-layout technique, called *dumbbell–stick diagrams* [9].

#### (i) Dumbbell–stick diagram

**Figure 7.**
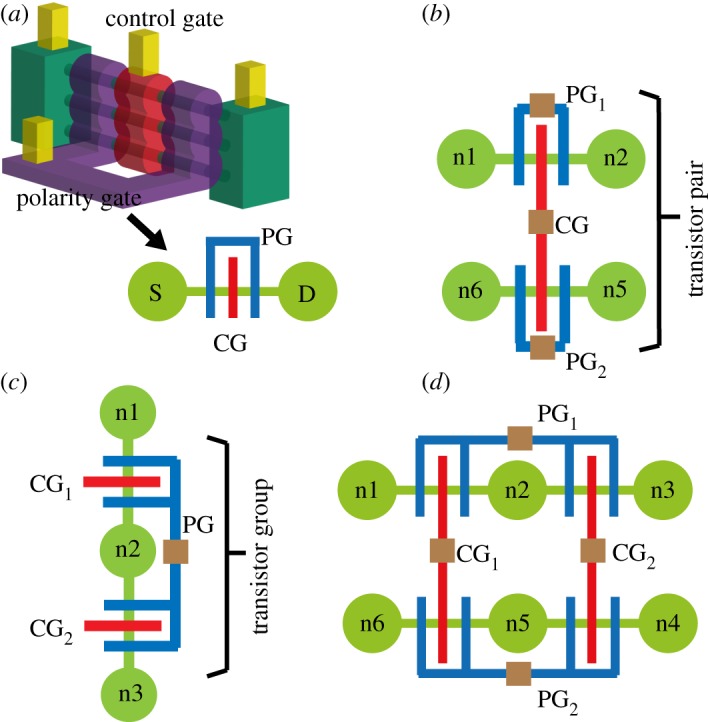
Dumbbell–stick diagram (*a*), transistor pairing (*b*), transistor grouping (*c*) and logic tile (*d*).

Similar to the CMOS stick diagrams [23], *dumbbell–stick diagrams* abstract the topology of logic gates with DG FETs technology. They are a convenient means for designing compact layouts and for minimizing the cell routing complexity. Figure 7*a* shows the dumbbell–stick diagram and how it is inspired by the physical shape of the device. The suspended SiNWs between the source and drain contacts form the basic dumbbell. The CG and the PG constitute the sticks. From this representation, we introduce the notion of transistor pairing and transistor grouping. *Transistor pairing* (figure 7*b*) helps in aligning the CGs of the complementary transistors in the pull-up and pull-down networks, whereas with *transistor grouping* (figure 7*c*) PGs of adjacent transistors are connected together. A logic tile is defined as an array of transistor pairs, with contiguous S/D pairs. Pairing and grouping reduce the number of *input* pins to the tile. A *tile*, consisting of two transistor pairs grouped together, is depicted in figure 7*d*. This simple tile is very effective in realizing logic primitives.

#### (ii) Layout technique for simple unate logic gates

**Figure 8.**
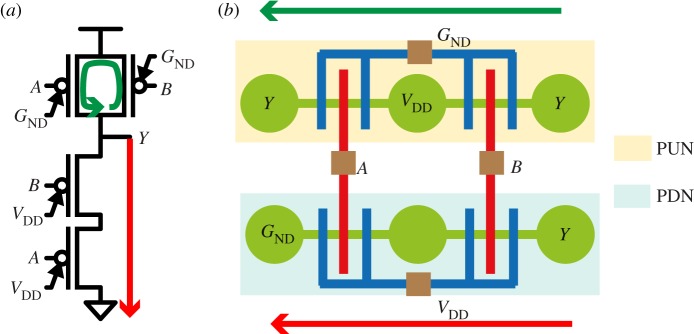
Schematic of a static two-input NAND gate (*a*) and its equivalent dumbbell–stick diagram (*b*).

Unate logic functions (e.g. NAND, NOR, AOI, etc.) with controllable-polarity devices are obtained by biasing the PGs of the *pull-up-network* (PUN) and *pull-down-network* (PDN) to *G*_ND_ and *V*_DD_, respectively. Hence, all the transistors in the PUN (and PDN) are grouped together (i.e. PGs of the stacked transistors are connected together). The personalization of the tile is reminiscent of the methods used for CMOS cells, which determine an optimum sequence of pairs with a minimum number of gaps [24]. Figure 8*a* shows an example of a two-input NAND gate with the PGs biased to either *G*_ND_ or *V*_DD_. Figure 8*b* shows its equivalent dumbbell–stick diagram.

#### (iii) Layout technique for simple binate logic gates

**Figure 9.**
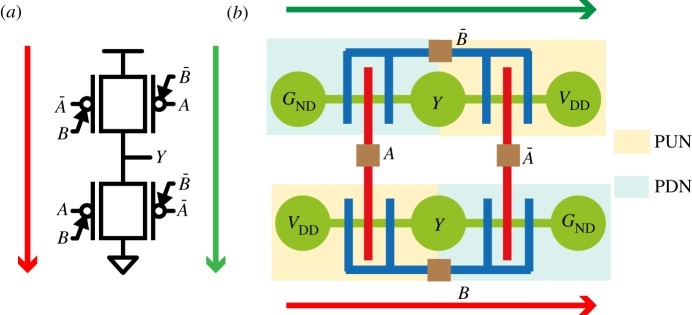
Schematic of a static two-input XOR gate [7] (*a*) and its equivalent dumbbell–stick diagram (*b*).

In the case of binate functions such as the XORs, the PGs in the PUN (and PDN) cannot be grouped, because they require independent inputs. An efficient implementation of a two-input XOR is shown in figure 9, where gates with similar polarity are grouped together to reduce routing. From the dumbbell–stick diagram, we can observe that the PUN and PDN are placed next to each other, which is possible with DG-SiNWFET technology as the transistors are field controlled to make them *p*-type or *n*-type. More complex cell designs have been proposed which leverage upon embedded XOR functionality of DG FETs [7,25,26].

#### (iv) Layout technique for sequential elements

**Figure 10.**
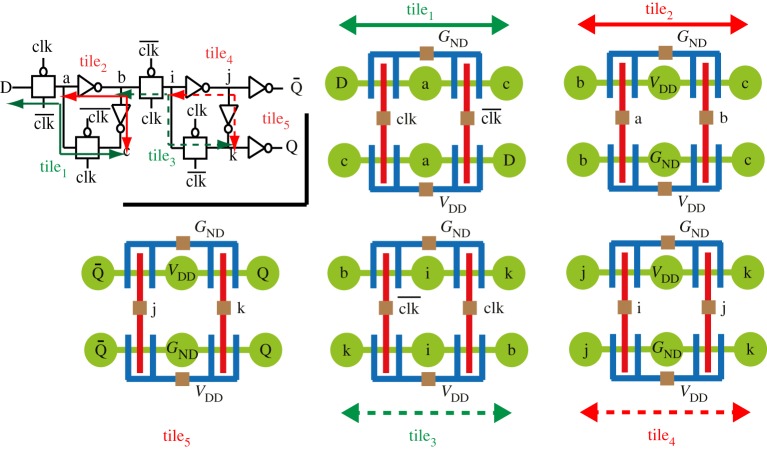
D flip–flop mapped on a regular set of tiles [19].

Sequential elements can still be efficiently mapped onto a set of tiles. Indeed, sequential elements often embed transmission gates that can be grouped together. Figure 10 illustrates a *D flip–flop* (DFF) mapped onto an array of tiles. In this implementation, we can observe that the two transmission gates in the master (slave) stage are physically mapped onto tile_1_ (tile_3_), efficiently compacting the overall mapping of the circuit. The inverters in the master, slave and output stages of the DFF are mapped onto tile_2_, tile_4_ and tile_5_, respectively. The inverting stage of the clock signal is not depicted.

## 4. Logic synthesis

Here, we summarize models and methods for performing effectively logic synthesis and mapping into an SoT.

Transistors with controllable polarity intrinsically embed the XOR logical connective and thus enable the realization of XOR operators with the same ease as NAND/NORs. The original logic synthesis methods [27–29], which are the basis for current commercial tools, use NAND/NOR representations and tend to be less effective for XOR-rich circuits, such as arithmetic operators and data paths. Other methods (e.g. BDS [30]) use *binary decision diagrams* (BDDs) to fully represent, manipulate and decompose logic functions. Thanks to the advantageous BDD-based XOR-decomposition techniques, BDS efficiently synthesizes XOR-intensive circuits. In the following, we show a formalism that is directly applicable to logic circuits to be implemented with XOR primitives, such as those based on DG-SiNWFETs. In particular, we introduce a novel BDD extension, called *biconditional binary decision diagrams* (BBDDs), that presents the advantage of directly supporting the behaviour of DG-SiNWFETs. Such a representation is canonical and demonstrates powerful properties when coupled to one-pass synthesis methodologies.

### (a) Biconditional binary decision diagrams

This section summarizes BBDDs. First, it presents the core logic expansion that drives BBDDs. Then, it gives ordering and reduction rules that make *reduced and ordered BBDDs*(ROBBDDs) canonical. A detailed description is given in [31].

#### (i) Biconditional expansion

In standard BDDs, each *non-terminal* node represents a Shannon expansion:

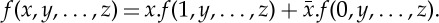
In BBDDs, the Shannon expansion is replaced by the *biconditional expansion:*

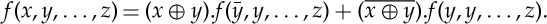
Note that the *biconditional expansion* is a special case of the (*x*_*i*_,*p*)-decomposition in [32] that extends the Shannon expansion. Note that only functions with two or more variables can be decomposed by a biconditional expansion. Indeed, in single variable functions, the XOR and XNOR terms cannot be computed. In such a condition, the biconditional expansion of a single variable function reduces to a Shannon expansion by setting the second variable *y* to logic 1. With this boundary condition, any Boolean function can be fully represented in terms of biconditional expansions.

#### (ii) Biconditional binary decision diagram structure and ordering

A BBDD is a BDD driven by the *biconditional expansion* in place of Shannon's expansion. Each non-terminal node in a BBDD has the branching condition *biconditional* on two variables. We call these two variables the *primary variable* (PV) and the *secondary variable* (SV).

**Figure 11.**
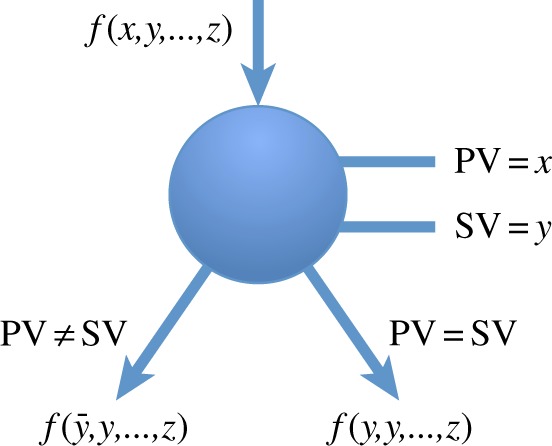
BBDD *non-terminal* node [31]. (Online version in colour.)

An example of a BBDD *non-terminal* node is provided in figure 11. We refer hereafter to PV =SV and PV=SV edges in a BBDD node simply as the ≠-edges and=-edges, respectively.

To achieve OBBDDs, a variable order must be imposed for PVs and a rule for the other variables assignment must be provided. We use the following *chain variable order* (CVO) to address this task. Given a Boolean function *f* and an order *π*=(*π*_0_,*π*_1_,…*π*_*n*−1_) of the inputs, PVs and SVs are ordered as

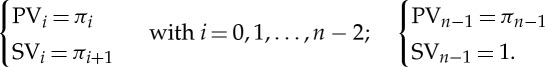
Note that if we swap *π*_*i*_ with *π*_*j*_ in the initial order *π*, owing to some reordering operation, this simply translates through the CVO as PV_*i*_ exchanged with PV_*j*_ and SV_*i*−1_ with SV_*j*−1_.

*Example:* from *π*=(*π*_0_,*π*_1_,*π*_2_), the corresponding CVO ordering is obtained by the following method. First, PV_0_=*π*_0_, PV_1_=*π*_1_ and SV_0_=*π*_1_, SV_1_=*π*_2_ are assigned. Then, PV_2_=*π*_2_ and SV_2_=1. The consecutive ordering by pairs (PV_*i*_, SV_*i*_) is thus ((*π*_0_,*π*_1_),(*π*_1_,*π*_2_),(*π*_2_,1)).

The CVO is a key factor enabling unique representation of ordered biconditional decision structures. We refer to ordered binary biconditional decision structures as BBDDs ordered by the CVO.

#### (iii) Biconditional binary decision diagram reduction

As in the case of OBDDs, also OBBDDs can be reduced to improve the representation efficiency, according to a set of rules. The straightforward extension of OBDD reduction rules [4] to OBBDDs corresponds to the iterated merging of isomorphic subgraphs.

Moreover, the OBBDD can be further reduced by eliminating levels with no nodes. Last, subgraphs that represent functions of a single variable can be collapsed into a single BDD node. Reduced OBBDDs are canonical [31].

### (b) One-pass logic synthesis

*One pass synthesis* (OPS) [33] is a logic synthesis methodology where logic optimization and technology mapping phases are combined in a single step carried out through a common data structure, e.g. BDDs. To target XOR-rich functions, we use BBDDs as data structure.

**Figure 12.**
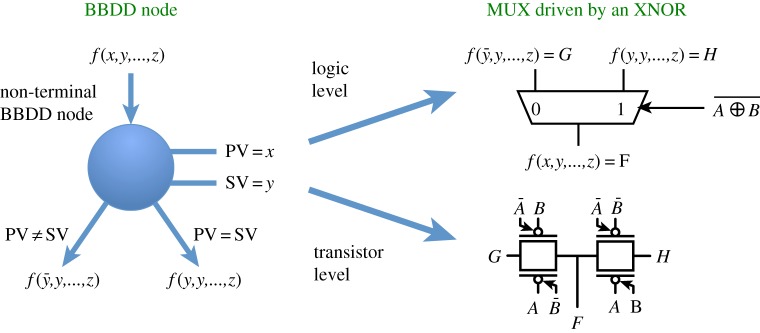
BBDD node corresponding logic gate and realization in ambipolar and CMOS technologies [31]. (Online version in colour.)

In BBDD-based OPS, logic optimization corresponds to the ROBBDD construction. Note that most of the algorithms for ROBDD construction, e.g. BUILD, APPLY [34], etc., can be adapted to ROBBDDs, hence to support the *biconditional expansion*in place of Shannon's expansion. Standard dynamic variable reordering algorithms can be applied also with the CVO (figure 12).

## 5. System-level design issues

The combination of the DG-SiNWFETs technology and BBDS-based synthesis can be applied to the design of both data path and control circuits. In particular, it enables the compact impact implementation of arithmetic functions and opens novel horizons in terms of testing and online fault detection.

### (a) Compact arithmetic operators

**Figure 13.**
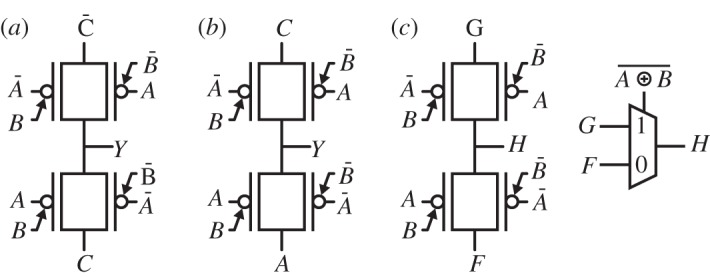
Transmission-gate three-input XOR (*a*), three-input majority logic gate (*b*) and generalized arithmetic gate (MUX-XNOR) (*c*).

DG-SiNWFETs enable the efficient design of parity circuits. Besides the efficient full-swing four-transistor XOR gate realization, shown in figure 4, a three-input XOR realization [26] leverages pass-transistor logic, as depicted in figure 13*a*. Note that in static CMOS, the same gate has 10 devices in place of 4 here [20].

Inspired by this last structure, a four-transistor three-input majority logic gate [35] is shown in figure 13*b*. This gate relies on the pass-transistor implementation of the MAJ(*A*,*B*,*C*) function rewritten as

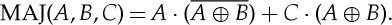
Note that in static CMOS, the same gate has 10 devices in place of 4 [20]. Moreover, the four DG-SiNWFETs configuration (of figure 13*a*) can be generalized to the MUX-like structure depicted in figure 13*c*. Its functionality corresponds to a multiplexer driven by an XNOR operation between *A* and *B*, selecting between two external signals *F* and *G*. With different assignments of *F* and *G*, it is possible to implement three-input MAJ(*F*=*A*, *G*=*C*), three-input MIN(*F*=*A*′, *G*=*C*′), three-input XOR(*G*=*C*′, *F*=*C*) and two-input XOR(*G*=1, *F*=0) logic gates. Therefore, this four-transistor structure can be seen as a generalized arithmetic gate.

**Figure 14.**
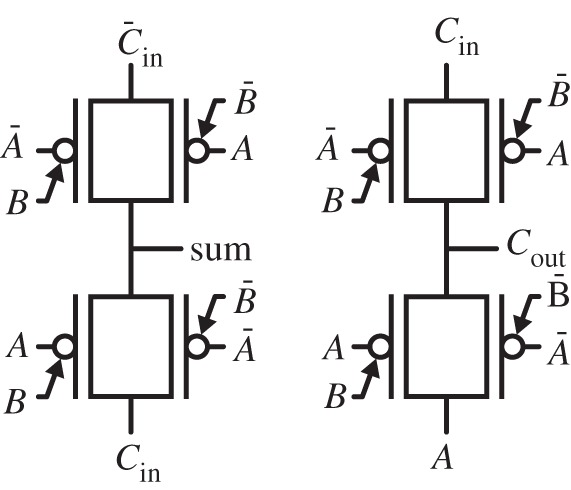
Full-adder implementation with eight controllable polarity devices.

The *full-adder* (FA) is a widely used arithmetic circuit that supports the addition of two binary numbers. It is represented by the following three-input two-output logic function:

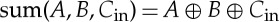
and

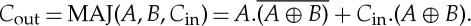
Controllable polarity transistors offer an advantageous implementation for both the *sum* and *C*_*out*_ functions using two generalized arithmetic gates. Therefore, the full-adder is competitively realized by eight devices, input inverters apart, as depicted by figure 14. The corresponding static (transmission gate) CMOS version has 28 (14) transistors [20].

### (b) Self-checking computation

**Figure 15.**
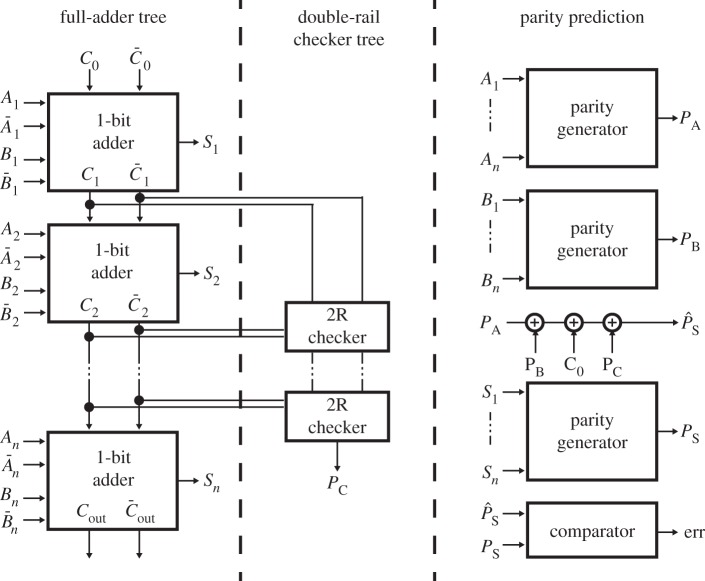
Self-checking *n*-bit adder using carry-checking parity-prediction scheme [36].

Among online testing strategies, self-checking circuits offer an efficient way of testing circuits without adding redundant voter circuitry such as in *triple modular redundancy* [36]. The most used self-checking technique is the parity prediction scheme [37]. Parity computation relies largely on the XOR operation, and therefore its implementation with the DG-SiNWFET technology can be fairly effective. The design of a self-checking ripple-carry adder has been introduced in [35] and is shown in figure 15.

The adder includes one-bit adders with complemented carry, double-rail checkers and parity generation trees. The complemented carry can be included within the existing FA structure, thanks to a compact minority operator. Indeed, only four extra transistors are required, whereas static CMOS design style needs 12 extra transistors. The parity-generation tree includes cascaded compact two-input XORs. Unfortunately, the compact four-transistor XOR implementation enabled by DG-SiNWFETs does not provide the fault-secure property. Indeed, in the case of a fault on the PGs, there exist some conditions where all the transistors take the same polarity, therefore leading to undetermined levels at the output. For this reason, in [35], a few parts of the circuit (the double-rail checkers) are still implemented using a traditional static CMOS implementation to guarantee the self-checking property. Nevertheless, the use of DG-SiNWFETs opens new opportunities also for fault-tolerant architectures.

## 6. Conclusion

We have presented here a complete design framework for nanoelectronic computational systems that leverage DG-SiNWFET technology. This framework includes semiconductor process development, device and circuit design, models and design tool research as well as architecting overall systems. In particular, we have shown the synergy of research results coming from novel device fabrication with circuit and architectural design. This research aims at achieving scalable arrays of nanodevices within regular arrangements, as a way to mitigate wiring variability. Last but not least, we have shown the challenges in design automation for nanotechnologies at various levels of abstraction.
